# Toward bioproduction of oxo chemicals from C_1_ feedstocks using isobutyraldehyde as an example

**DOI:** 10.1186/s13068-022-02178-y

**Published:** 2022-08-09

**Authors:** Liwei Guo, Lichao Sun, Yi-Xin Huo

**Affiliations:** Key Laboratory of Molecular Medicine and Biotherapy, School of Life Science, Beijing Institute of Technology, No. 5 South Zhongguancun Street, Beijing, 100081 People’s Republic of China

**Keywords:** Oxo chemicals, Isobutyraldehyde, Bioproduction, C_1_ feedstocks

## Abstract

Oxo chemicals are valuable chemicals for synthesizing a wide array of industrial and consumer products. However, producing of oxo chemicals is predominately through the chemical process called hydroformylation, which requires petroleum-sourced materials and generates abundant greenhouse gas. Current concerns on global climate change have renewed the interest in reducing greenhouse gas emissions and recycling the plentiful greenhouse gas. A carbon–neutral manner in this regard is producing oxo chemicals biotechnologically using greenhouse gas as C_1_ feedstocks. Exemplifying isobutyraldehyde, this review demonstrates the significance of using greenhouse gas for oxo chemicals production. We highlight the current state and the potential of isobutyraldehyde synthesis with a special focus on the in vivo and in vitro scheme of C_1_-based biomanufacturing. Specifically, perspectives and scenarios toward carbon– and nitrogen–neutral isobutyraldehyde production are proposed. In addition, key challenges and promising approaches for enhancing isobutyraldehyde bioproduction are thoroughly discussed. This study will serve as a reference case in exploring the biotechnological potential and advancing oxo chemicals production derived from C_1_ feedstocks.

## Introduction

Climate change caused by greenhouse gas emissions remains a global threat, which has raised worldwide attention. A non-negligible source of greenhouse gas emission is the chemical manufacturing of petroleum-based oxo chemicals, including oxo aldehydes, oxo acids, and oxo alcohols. These chemicals are critical platform chemicals that have been widely used in the manufacture of a variety of industrial and consumer products [1]. Bio-manufacturing serves as a carbon-neutral route by utilizing bio-catalysts for producing highly valued products from low-cost feedstocks. Compared with sugar-based feedstocks and non-food lignocellulose, greenhouse gas such as carbon dioxide (CO_2_) and methane are emerging as attractive C_1_ feedstocks due to their ready availability, low cost and the benefits to slow down global warming [2]. In the past few years, advances in biotechnologies such as synthetic biology, metabolic engineering, and adaptive evolution have accelerated the progress in the fixation and assimilation of C_1_ feedstocks in native and synthetic C_1_-utilizing organisms [3–7]. These impressive efforts have renewed the interest in converting C_1_ feedstocks to high-value organic chemicals [8–13]. Nevertheless, biomanufacturing of oxo chemicals using C_1_ feedstocks is still scarce.

In this review, we highlight the current state and conversion potential of C_1_ feedstocks to oxo chemicals using isobutyraldehyde as an example. Isobutyraldehyde is a representative oxo aldehyde that serves as a platform chemical for producing a large portfolio of bioproducts including alcohols, acids and alkanes. Specifically, we summarize recent advances for isobutyraldehyde synthesis, prospects for the in vivo and in vitro conversion of C_1_ feedstocks to isobutyraldehyde, discuss the challenges, and propose the engineering approaches for enhancing isobutyraldehyde production. This work aims at providing a reference case for the future advancement of oxo chemicals production derived from C_1_ feedstocks.

## Potential market

World consumption of oxo chemicals surpassed 10 million metric tons per year with an average annual growth rate of over 3%. The majority market is occupied by the global major chemical manufacturers, such as Eastman Chemical Company, BASF, The Dow Chemical Company, BAX Chemicals BV, ExxonMobil Chemical Company, and OXEA Group. Among all the oxo chemicals, approximately 77% of the world’s consumption were the propylene-derived n-butyraldehyde and isobutyraldehyde [14]. As an isoform of butyraldehydes, isobutyraldehyde serves as a platform for producing a plethora of bioproducts, including isobutanol, neopentyl glycol, isobutyric acid, isobutyl acetate, isobutylidene diurea, and methyl isoamyl ketone. Isobutyraldehyde has been applied in the manufacture of a multitude of products including solvents, oil additives, paints & coatings, plasticizers, herbicides, fertilizers, fragrances, and flavoring agents, pharmaceuticals, and cosmetics [15]. Currently, isobutyraldehyde has a growing global market at a magnificent CAGR（Compound Annual Growth Rate) and is highly demanded in the area of construction, food, pharmaceutical, cosmetics.

## Chem vs bio-process

**Fig. 1:**
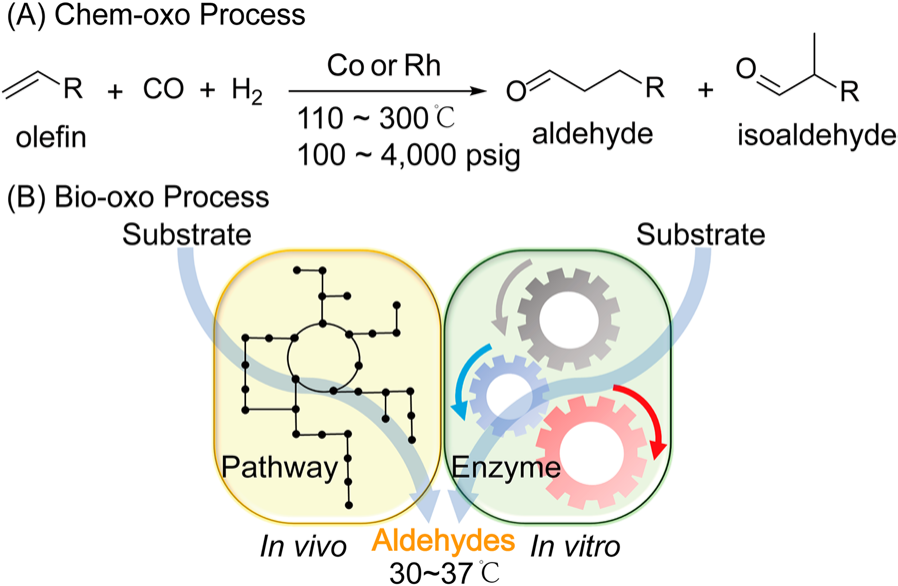
Comparison between chemical oxo process and biological oxo process. **A** Oxo aldehydes are manufactured from the hydroformylation of olefin and synthesis gas, also known as the chemical oxo (chem-oxo) process. The chem-oxo process is driven by either cobalt (Co) hydrocarbonyl catalyst or triphenylphosphine rhodium (Rh) hydrocarbonyl catalyst and requires harsh conditions including high pressure and high temperature. **B** Oxo aldehydes are produced using either metabolically engineered microorganisms (in vivo) or enzyme catalysts (in vitro), also termed as biological oxo (bio-oxo) process. During the bio-oxo process, the C–C bond was formed to produce pure aldehydes from renewable resources under mild operation conditions

Isobutyraldehyde can be manufactured from the hydroformylation of propylene and synthesis gas, also known as the chemical oxo (chem-oxo) process (Fig. 1A). This catalyst-driven chemical process involves the addition of a formyl group and a hydrogen atom to propylene under pressure. Chemical production processes adopt one of two catalytic processes. One process involves a cobalt (Co) hydrocarbonyl catalyst-driven process, which utilizes HCo(CO)_4_ to react propylene with syngas at 110–170 °C and 1500–4000 psig, producing n-butyraldehyde and isobutyraldehyde at a ratio of 2:1–4:1. The other process uses a triphenylphosphine rhodium (Rh) hydrocarbonyl catalyst HRh(CO)(Ph_3_)_3_ to promote the reaction of propylene and syngas at 110 °C and 100 –300 psig, achieving approximately 8:1 to 12:1 ratio of n-butyraldehyde: isobutyraldehyde [16]. The two chem-oxo processes utilize high temperature and pressure conditions, thus requiring significant energy consumption. In addition, the products of these two processes are the isomer mixture of butyraldehyde, which need energy-intensive downstream processes such as fractional distillation processes to separate isobutyraldehyde from the mixture. Another problem of the chem-oxo process is the azeotropic phenomenon resulting from the close vapor pressures of isobutyraldehyde (17 kPa at 20 °C) and n-butyraldehyde (12 kPa at 20 °C). Beyond these, volatile prices of raw materials and natural gas supply are also the key restraints to the isobutyraldehyde market.

The increasing value of isobutyraldehyde and the constraints of the chem-oxo process make isobutyraldehyde a highly demanded chemical compound with a rising price. Preliminary studies have achieved isobutyraldehyde production using the metabolically engineered microorganisms or enzyme catalysts via the biological oxo (bio-oxo) process, during which the C–C bond was formed to produce pure isobutyraldehyde from renewable resources under mild reaction conditions (30–37 °C, 0 psig) (Fig. 1B) [17–19]. The microbial process utilizes the energetically gas-stripping strategy for efficient in situ product recovery taking advantage of the low vapor pressure and boiling point of isobutyraldehyde. The collection and purification process has the additional advantage of alleviating toxicity and inhibitory effects due to the reduced exposure of the strain or enzymes to the product [20]. The availability of bio-isobutyraldehyde would allow for the direct replacement of the chem-oxo process and be the most straightforward way to bio-based derivatives.

## Microbial production of isobutyraldehyde

Isobutyraldehyde can be synthesized from glucose through the branched-chain amino acid pathway in microorganisms [21]. Isobutyraldehyde production hijacks the well-established isobutanol pathway by neglecting the last step, where the isobutyraldehyde reductase (IBR) catalyzes the conversion of isobutyraldehyde to isobutanol. Through the overexpression of heterologous, 2-keto-acid decarboxylase (KivD) from *Lactococcus lactis*, isobutyraldehyde can be produced from 2-ketoisovalerate (KIV), a precursor that is endogenously generated via valine metabolism. The titer of isobutyraldehyde has been enhanced by elevating the level of KIV via the overexpression of the upstream enzymes, and the endogenous conversion of isobutyraldehyde to isobutanol has been minimized by deleting alcohol dehydrogenases (ADHs) and aldehyde reductases (ALRs), resulting in 35 g/L of isobutyraldehyde production in *E. coli* with a productivity of 0.29 g/L/h [22] (Table 1). By screening from a pool of 44 candidates that were annotated as NAD(P)H- or FAD-dependent reductase or dehydrogenase in *E. coli* genome database, five additional genes (*yahK*, *dkgA*, *ybbO*, *gldA*, *and yghA*) exhibiting obvious ALR activities were confirmed. Deletions of all proven ALRs finally resulted in a 90–99% reduction in ALR activity and increased production of aldehydes along with minor alcohols (2–15%), showing the importance of the serial and combined gene deletion strategy in isobutyraldehyde production [23].

**Table 1:** Production of isobutyraldehyde and isobutanol using different substrates

Substrate	Host	Isobutanol			Isobutyraldehyde		
		Scale	Production	Refs.	Scale	Production	Refs.
Glucose	*E. coli*	Flask	22 g/L	[24]	Flask	35 g/L	[22]
Glucose	*E. coli*	Bioreactor	56 g/L	[25]			
Glucose	*B. megaterium*	Test tube	0.3 g/L	[26]			
Glucose	*B. subtilis*	Bioreactor	3.83 g/L	[27]			
Glucose	*C. glutamicum*	Bioreactor	13 g/L	[28]			
Glucose	*S. cerevisiae*	Flask	1.62 g/L	[29]			
Glucose	Cell-free system	Bioreactor	275 g/L	[30]			
CO_2_	*S. elongatus*	Bottle	0.45 g/L	[18]	Bottle	1.1 g/L	[18]
					Flask	0.05 mmol/gDW/h	[31]
					Flask	1.2 mmol/L	[32]
CO_2_	*R. eutropha*	Bioreactor	0.09 g/L	[33]			
Lignocellulose	*C. crenatium*	Bottle	5.61 g/L	[34]			
Cellulose	*C. cellulolyticum*	Not specified	0.66 g/L	[35]			
Fructose	*R. eutropha*	Flask	0.27 g/L	[36]			

## In vivo framework converting C_1_ feedstocks to isobutyraldehyde

### Producing isobutyraldehyde from CO_2_

Incorporating atmospheric CO_2_ into chemicals by either photosynthetic bacteria or genetically engineered heterotrophs is a promising solution to slow down climate change and thus will contribute to the green economy [37]. *Synechococcus elongatus* is a commonly used cyanobacterium and could tolerate isobutyraldehyde as high as 750 mg/L. Through genomic integration of the isobutyraldehyde pathway and overexpression of ribulose 1,5-bisphosphate carboxylase/oxygenase (RuBisCo) in *S. elongatus* PCC7942, isobutyraldehyde with a titer of 1.1 g/L and a production rate of 6.23 mg/L/h was produced over 8 days by applying in situ product removal system [18]. The endogenous activity of ADHs was not detectable in *S. elongatus*, showing the potential of this strain in the continuous production of isobutyraldehyde. Despite relatively low titer and productivity, this work is a proof-of-concept study showing the in vivo conversion of CO_2_ to isobutyraldehyde.

Isotopically nonstationary ^13^C metabolic flux analysis (INST-MFA) is a versatile method for assessing the central fluxes and metabolite profiling of microorganisms [38, 39]. Flux bottlenecks of isobutyraldehyde-producing cyanobacteria has been identified through INST-MFA. By comparing the pathway fluxes of wild-type *S. elongatus* PCC7942 and the isobutyraldehyde-production strain SA590, the pyruvate kinase (PK) bypass pathway was verified as a bottleneck in the conversion of PEP to pyruvate, guiding rational engineering for higher production of isobutyraldehyde via overexpressing PK pathway genes [31]. Further systematic analysis using INST-MFA verified the sensitivity of isobutyraldehyde production toward the flux perturbations of pyruvate nodes and demonstrated the positive effects of attenuating flux into the competing pathway in the improvement of titer and production rate [32].

Currently, the productivity of isobutyraldehyde in cyanobacteria is far below the level of 35 g/L mainly due to the low efficiency of CO_2_ fixation and the limited synthetic biology tools for engineering [22]. Many approaches have been proposed and adopted in cyanobacteria for achieving efficient CO_2_ fixation, including overexpression of genes of the Calvin–Benson–Bassham (CBB) cycle and optimization of the CO_2_ fixation enzymes [3]. However, the CBB cycle has several innate limitations, such as the kinetic inefficiency of RuBisCO and the carbon loss during the fixation process. Improvement of RuBisCO is practically challenging and limited progress has been reported. Alternative solutions have been proposed such as reducing carbon loss via coupling CBB cycle with a synthetic malyl-CoA-glycerate (MCG) pathway, which allows the fixation of an additional CO_2_ equivalent for the synthesis of extra acetyl-CoA [25]. Nevertheless, CBB cycle that suffers from low ATP-efficiency and low conversion rate may not be an ideal solution from the economical perspective.

**Fig. 2:**
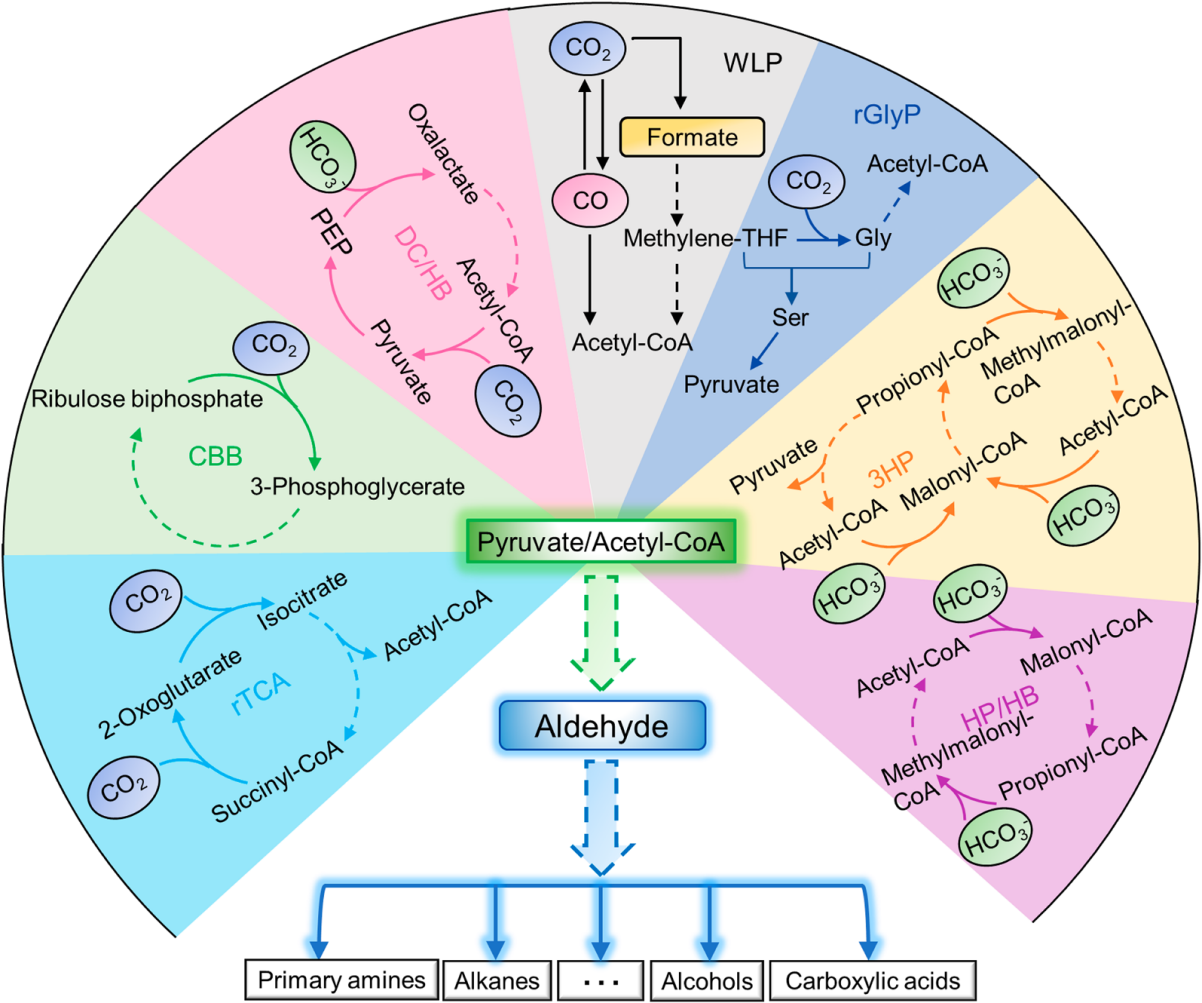
In vivo framework converting C_1_ feedstocks to aldehydes. Seven in vivo pathways converting CO_2_ to either pyruvate or acetyl-CoA might be employed to form aldehydes, which are important platform chemicals for value-added products such as primary amines, alkanes, and alcohols, etc. These pathways include reductive TCA (rTCA) cycle, Calvin–Benson–Bassham (CBB) cycle, dicarboxylate/4-hydroxybutyrate (DC/HB) cycle, Wood–Ljungdahl (reductive acetyl-CoA) pathway (WLP), reductive glycine pathway (rGlyP), 3-hydroxypropionate (3-HP) bicycle and 3-hydroxypropionate/4-hydroxybutyrate (HP/HB) cycle. Each pathway is distributed within a sector with a color of the background. The dashed line indicates steps consisting of multiple reactions while the solid line indicates one step

Apart from the CBB cycle, six other CO_2_ fixation pathways have been validated in vivo, including five native ones, Wood–Ljungdahl (reductive acetyl-CoA) pathway (WLP), reductive TCA (rTCA) cycle, dicarboxylate/4-hydroxybutyrate (DC/HB) cycle, 3-hydroxypropionate/4-hydroxybutyrate (HP/HB) cycle, 3-hydroxypropionate (3-HP) bicycle, and an artificial one, reductive glycine pathway (rGlyP) (Fig. 2). Key factors such as energy source and catalytic efficiency have been comprehensively analyzed for evaluating the efficiency of these pathways [40]. It would be interesting to introduce the energy-efficient CO_2_ fixation pathways into isobutyraldehyde production strain to achieve higher titers through the combination with an effective product removal system. To this end, each C_1_-pathway was employed to assemble with the isobutyraldehyde pathway, and the enzyme numbers, ATP cost, NAD(P)H equivalents were calculated individually (Table 2).

**Table 2:** Enzyme numbers and cofactor equivalents for isobutyraldehyde production from C_1_ feedstocks

Substrate	C_1_-Pathway	Intermediate	Enzyme number	ATP cost	NAD(P)H equivalents	Refs.
CO_2_	WLP	acetyl-CoA	12	2	11	[47]
CO_2_	rGlyP	pyruvate	11	4	11	[42, 48]
CO_2_	rTCA cycle	acetyl-CoA	13	4	11	[49, 50]
CO_2_ HCO_3_^−^	DC/HB cycle	acetyl-CoA	18	6	11	[51]
CO_2_ HCO_3_^−^	HP/HB cycle	acetyl-CoA	20	8	11	[52, 53]
HCO_3_^−^	3-HP bicycle	pyruvate	22	10	11	[54, 55]
CO_2_	CBB cycle	GAP	21^a^	14^a^	11^a^	[56]
CO_2_	ASAP	pyruvate	13^a^	− 2^a^	11^a^	[13]

All calculations are based on converting CO_2_ equivalents to isobutyraldehyde via pyruvate. NAD(P)H equivalents refer to the reducing power generated from NADH, NADPH, ferredoxin, H_2,_ or H_2_O.

WLP: Wood–Ljungdahl pathway, rGlyP: reductive glycine pathway, rTCA cycle: reductive TCA cycle, DC/HB cycle: dicarboxylate/4-hydroxybutyrate cycle, HP/HB cycle: 3-hydroxypropionate/4-hydroxybutyrate cycle, 3-HP bicycle: 3-hydroxypropionate bicycle, CBB cycle: Calvin–Benson–Bassham cycle, ASAP: artificial starch anabolic pathway.

^a^Here, we assume that the CBB cycle and ASAP are employed to convert CO_2_ to glyceraldehyde-3-phosphate (GAP), then a few steps of the glycolysis pathway are utilized to convert GAP to pyruvate, which will be further used to produce isobutyraldehyde

Among these pathways, rGlyP, CBB cycle, and 3-HP bicycle can directly generate pyruvate, which is readily converted to isobutyraldehyde. No ATP is consumed during the formation of isobutyraldehyde from pyruvate, thus the total ATP cost of the reassembled pathways is equal to the ATP quantities consumed by the C_1_-pathways solely. The rest, including WLP, rTCA, DC/HB cycle, and HP/HB cycle, produce acetyl-CoA as an output. To condense acetyl-CoA and CO_2_ into pyruvate, a four-step carbon fixation cycle catalyzed by pyruvate carboxylase, oxaloacetate acetylhydrolase, acetate-CoA ligase, and pyruvate synthase (POAP cycle) was recently established [41]. By reassembling with pyruvate synthase or other enzymes that convert acetyl-CoA to pyruvate, C_1_-pathways including WLP, rTCA, DC/HB cycle, and HP/HB cycle can be employed for producing isobutyraldehyde [42]. Accordingly, the ATP cost of the reassembled pathways included the ATP consumptions of the C_1_-pathways and an additional ATP for generating one molecule of pyruvate from acetyl-CoA.

It should be noted that NAD(P)H consumption equals the reducing power required for changing the valence state of carbon from the substrate to the product. Although six molecules of CO_2_ and/or HCO_3_^−^ are fixed, the net reaction is the reduction of four molecules for producing one molecule of isobutyraldehyde (C_4_H_8_O). Assuming hydrogen in isobutyraldehyde is always positively monovalent (1 +) and oxygen is always negative divalent (2-), then the four carbons together are negative hexavalent (6-). Because carbon has a valence of 4 in CO_2_ and HCO_3_^−^, 22 electrons will be required to reduce the four carbons (16 +) to that of isobutyraldehyde. Since every two electrons can be provided from one molecule of NAD(P)H, 11 NAD(P)H equivalents will meet the requirements for reducing the oxidation state of CO_2_. Therefore, no matter which pathway is used for converting CO_2_ to isobutyraldehyde, the NAD(P)H equivalents consumed should be the same, namely, 11.

Among these C_1_-fixation pathways, WLP is an outstanding candidate, which requires only two ATPs for isobutyraldehyde production. WLP-harboring strains such as acetogenic Clostridia have been successfully employed in ethanol and butanol production with high conversion efficiencies [43]; however, the transplantation of this pathway to non-C_1_ utilizers is still a difficult task due to the requirement of complex enzymes and the restriction to anaerobic conditions. In contrast, rGlyP consists of oxygen-tolerant, naturally existing, and efficient enzymes and shows limited overlap with central metabolism. rGlyP has been demonstrated effective in non-C_1_ utilizers, successfully mediating decent growth of *E. coli* and *Cupriavidus nector* on formate [6, 44]. The reassembled route of rGlyP and isobutyraldehyde pathway consists of the simplest steps and consumes only four ATPs, thus is a preferred choice for isobutyraldehyde synthesis. rTCA cycle is also an advantageous choice with fewer steps and ATPs cost. Although this pathway has not yet been reported for autotropic growth, it has been incorporated into non-C_1_ utilizers for improving malate production from glucose [45]. Considering alternative C_1_ substrates can be readily converted from CO_2_ closest to industrial scale, we may see a bright future for using these C_1_-pathways for biochemical production in engineered biotech hosts [46]. The rest C_1_-pathways have not yet been demonstrated in any biochemical production and are not suitable for isobutyraldehyde production due to the requirement of multiple steps and high ATP cost.

### Exploring various hosts for isobutyraldehyde synthesis

Beyond cyanobacteria, natural C_1_-utilizers such as lithoautotrophic *Ralstonia eutropha*, *Acetobacterium woodii*, and *Clostridium ljungdahlii* are also worth studying for producing isobutyraldehyde. By combining the electrochemical and microbial process, CO_2_ can be converted to energetic C_1_ compounds, such as formate and methanol [46]. The probability of the hybrid microbial electrosynthesis method has been verified in *R. eutropha* using an electro-bioreactor, yielding 0.85 g/L isobutanol [33]. Other than aerobic *R. eutropha*, the acetogenic *C. ljungdahlii* that grows well on syngas (CO + H_2_ + CO_2_ mixture) has also been engineered to produce isobutanol under autotropic conditions [57]. Regarding the relationship between isobutanol and isobutyraldehyde, these microbial hosts show promise in isobutyraldehyde production regardless of the energy demands for hydrogen cost [58]. Although the immaturity of engineering toolboxes for native C_1_-utilizers hindered the efficient knockout of multiple ADHs and ALRs currently, the rapid advancement of gene editing techniques paves the way toward future solutions. Using the electroporation-based CRISPR–Cas9 technique, an efficient genome editing method has been developed for *R. eutropha*, one of the hosts natively harboring the CBB cycle, leading to the editing of five genes with efficiencies ranging from 78.3 to 100% [59]. Therefore, it’s highly expected to realize editing of multiple ADHs and ALRs in the native C_1_-utilizers in the future.

In addition to employing C_1_-utilizers, constructing synthetic C_1_-utilizing pathways in mature biotechnological platforms is also gaining increasing attention in C_1_-based biomanufacturing areas [60]. Biotechnologically relevant organisms such as prokaryotic *E. coli*, eukaryotic *Pichia pastoris,* and *Yarrowia lipolytica* have been recently rewired to be capable of growing on formate, methanol or/and CO_2_, shedding light on the sustainable and low-cost synthesis of isobutyraldehyde in these industrial strains [4–7, 61, 62]. However, many challenges such as metabolite and cofactor imbalance, enzymes constraints, substrates and intermediates toxicity remain to be addressed to further take advantage of C_1_ metabolism for isobutyraldehyde synthesis.

### Toward carbon– and nitrogen–neutral isobutyraldehyde production

Currently, C_1_-based biomanufacturing mainly focus on achieving carbon neutrality. However, these processes require supplementation of reduced nitrogen sources for producing proteins and nucleic acids while generating high-nitrogen-containing residuals as byproducts, leading to dispersion of nitrogen on earth and an increase of toxic nitrous oxide (N_2_O) in greenhouse gas [63]. To address this issue, an ideal way is to utilize protein-based refinery to recycle the nitrogen source from the protein-rich residuals while realizing carbon–neutral biomanufacturing. Protein-based refinery is also termed as nitrogen–neutral amino acids refinery, which uses a deamination or transamination process to release carbon skeletons for chemicals production while recycling nitrogen in the form of ammonia [64]. Specifically, enzymes converting amino acids to the corresponding α-keto acids or tricarboxylic acid (TCA) cycle intermediates were introduced into the hosts to generate the precursors for chemical production. The feasibility of protein-based biorefinery was first demonstrated as a proof-of-concept by introducing LeuDH from *Thermoactinomyces intermedius* to *E. coli*, driving the transamination reactions and the conversion of proteins to isobutanol [65].

Because isobutyraldehyde is an upstream product of isobutanol, the system above could be applied for isobutyraldehyde production. In this regard, utilization of protein waste that are generated during C_1_-based isobutyraldehyde production toward additional synthesis is conceptually feasible and economically viable. Various transaminases and deaminases that catalyze amino acids to α-keto acid precursors are worth testing for this purpose [66, 67]. To be noted, the deamination process will release ammonia for the synthesis of glutamate and glutamine through the intrinsic ammonia assimilation pathway; therefore, it is necessary to disrupt the assimilation process to avoid the reincorporation of ammonia. Blocking ammonia reutilization will create a nitrogen limitation condition, because amino acids in the protein hydrolysates serve as a poor nitrogen source for cells. Such a condition is beneficial for sustaining the transcriptional activity of nitrogen-responsive promoters, which have been proved to be effective in facilitating robust protein-to-isobutanol conversion [68]. Such transcriptional machinery also holds promise in protein-based isobutyraldehyde production.

It is reported that protein-rich algae with less lipid content have high biomass productivity and contain large amounts of nitrogen and carbon [63]. Therefore, another scenario that could be applied to isobutyraldehyde production is utilizing C_1_-feedstock for biomass growth of protein-rich algal species, then harvesting and hydrolyzing the protein-rich biomass for isobutyraldehyde production. This may offer an economic and efficient production track for C_1_-based isobutyraldehyde manufacturing. Through metabolic engineering and process optimization, the above proposals can be cost-effective in achieving carbon– and nitrogen–neutral isobutyraldehyde production while maximizing the C_1_ fixation efficiency.

### General strategies for enhancing isobutyraldehyde production

Although isobutyraldehyde has achieved a decent titer in the g/L range using sugar-based feedstocks, hyper-production is limited due to several reasons. First, the inherent instability of isobutyraldehyde poses a considerable challenge for the biosynthesis. Isobutyraldehyde and nearly all the generated aldehydes are spontaneously and rapidly converted to the corresponding alcohols in microorganisms by endogenous ADHs and ALRs [69]. Second, the toxicity of aldehydes to microorganisms restricts large-scale biological production and remains a practical issue of academic and commercial interest [20]. Isobutyraldehyde shows a distinct extent of toxicity to different microorganisms. For example, 1 g/L of isobutyraldehyde decreased the cell growth of *E. coli* to 30% within 4 h and 10 g/L of isobutyraldehyde totally inhibited the growth [22]. The treatment of isobutyraldehyde at a concentration of 1 g/L abolished the cell growth of *S. elongatus* [18]. Third, the redox balance of the isobutyraldehyde pathway is difficult to be maintained during the long-term production due to the cell viability and complexity. Fourth, isobutyraldehyde is a volatile molecule and can be easily evaporated into the air during production. Other limiting factors include the chemically reactive property, which leads to the formation of biological compounds, such as isobutyric acid [21].

**Fig. 3:**
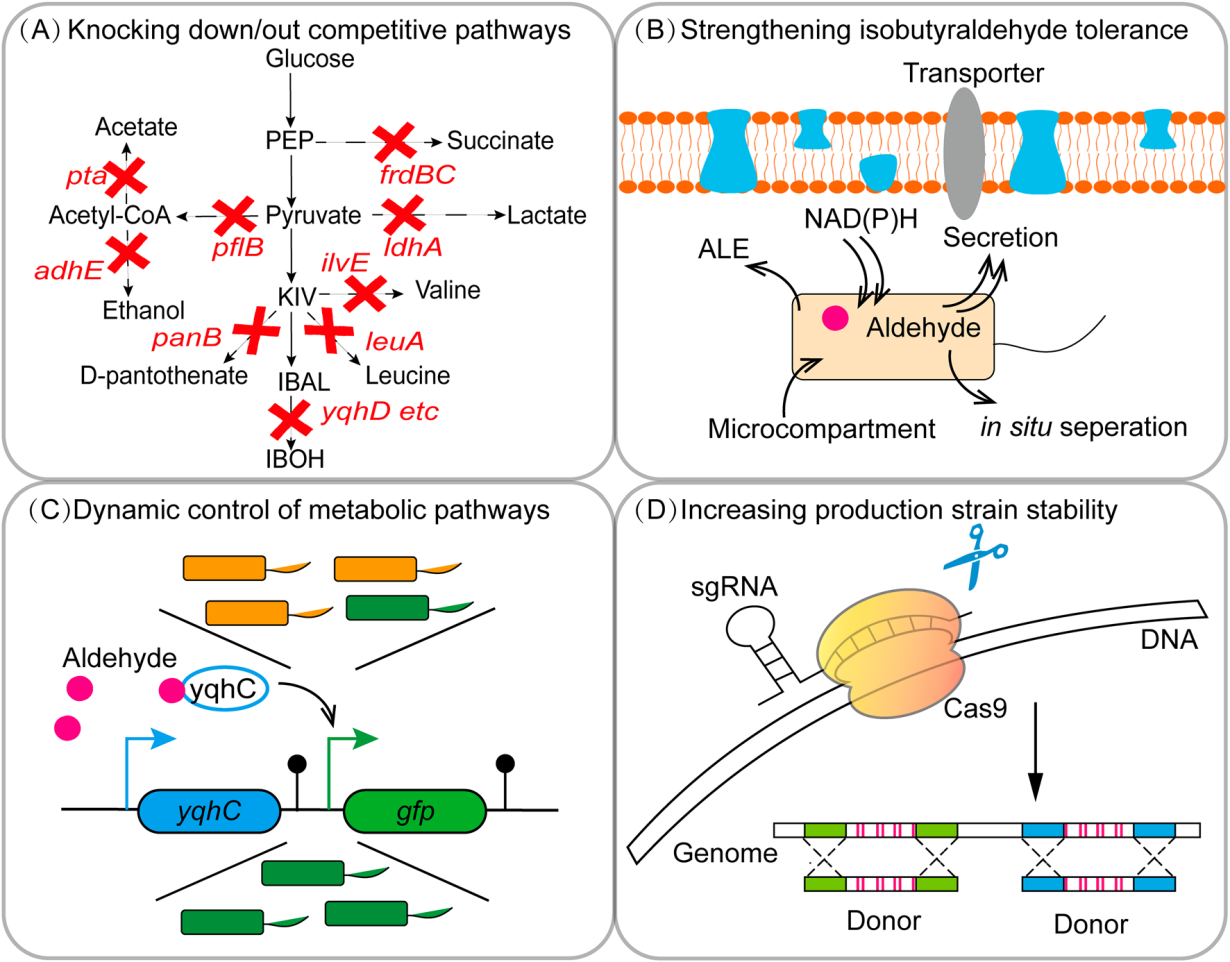
General engineering strategies for enhancing isobutyraldehyde production. **A** Knocking down/out the degradation and competitive pathways. **B** Strengthening isobutyraldehyde tolerance. **C** Dynamic control of metabolic pathways using the sensing system. **D** Increasing production strain stability via multicopy chromosomal integration of target pathways

Therefore, for isobutyraldehyde and other oxo chemicals with similar properties, achieving production on an industrial scale is attractive but challenging. Here, potential engineering strategies and fermentation optimization approaches are thoroughly explored. General engineering strategies include deleting genes of degradation and competing pathways, strengthening isobutyraldehyde tolerance, dynamic control of metabolic pathways and increasing strain stability (Fig. 3).

### Knocking down/out degradation and competitive pathways

*E. coli* has many enzymes such as YqhD showing ADH or ALR activities. Protein BLAST based on sequence similarity is a convenient and feasible tool to identify the potential ADHs and ALRs. It is easy to confirm the candidate activity toward isobutyraldehyde in vitro; however, the deletion of single or several genes may have a minor influence on the aldehyde production due to the redundancy of various ADHs and ALRs. With the development of CRISPR-assisted multiple genes editing techniques, the systematic gene deletion method that knock out multiple candidates simultaneously is conceptually desirable. This will facilitate the systematic genome-wide screening for genes annotated as reductase and dehydrogenase as well as genes predicted with “orphan” metabolic activities or known biochemical activities that are not currently assigned to genes in some or all organisms.

Further improvement in the production of isobutyraldehyde can be achieved by the deletion of competing pathways. For example, gene knockouts of the byproduct pathways, such as lactate (*ldhA*), acetyl-CoA (*pflB*), ethanol (*adhE*), acetate (*pta*), and succinate (*frdBC*) contribute to pools of cofactors and pyruvate. Gene knockouts of metabolic pathways, such as valine biosynthesis (*ilvE*), leucine biosynthesis (*leuA*), and D–pantothenate biosynthesis (*panB*), can reduce the loss of the precursor KIV (Fig. 3A).

### Strengthening isobutyraldehyde tolerance

The toxicity of isobutyraldehyde and other aldehydes has been a challenging issue in microbial aldehydes production and lignocellulose utilization [69]. To address this, mechanisms underlying aldehyde toxicity have been extensively studied [20, 70, 71] and general strategies alleviating the toxicity have been well-reviewed, including the reduction of aldehydes [72], the supply of NAD(P)H [73], the establishment of protein microcompartments [74], the engineering of protection and repair systems [75], the efficient secretion [76] and in situ separation [22]. While some tolerance mechanisms are specific to phenolic and cyclic aldehydes, tolerance engineering strategies based on these mechanisms might translate to aliphatic aldehydes including isobutyraldehyde. Considering the structural difference of phenolic and cyclic aldehydes, the research on tolerance mechanisms and tolerance engineering strategies of isobutyraldehyde might require additional efforts. Several transporters correlating to the enhanced tolerance of phenolic aldehydes, including ABC transporters, MFS transporters, and RND transporters, may serve as promising targets for future engineering of isobutyraldehyde production strains (Fig. 3B). An easy way to obtain the isobutyraldehyde-tolerant strain is to employ the adaptive laboratory evolution (ALE) strategy, which has been demonstrated effective in developing strains with high tolerance toward aldehydes [77]. The hotspots and valuable targets for mutant fitness could be explored via next-generation sequencing. Another strategy is to explore the potential of naturally phenolic aldehydes-tolerant microbial hosts, such as *Zymomonas mobilis* in the production of isobutyraldehyde.

### Dynamic control of metabolic pathways

A biosensor toward a specific molecule can transduce its concentration into gene expression changes, facilitating dynamic control of the metabolic network and fast screening of genetic variants with different metabolic designs [78]. Biosensor-based engineering has been a viable strategy for improving the production of the target molecule [79]. The sensor module generally consists of a transcriptional regulator that is responsive to the target molecule and a reporter gene driven by the corresponding cognate promoter. YqhD is the major ALR in *E. coli* and is expressed from the *yqhD-dgkA* operon [80]. YqhC is a transcriptional regulator that induces the *yqhD-dgkA* operon in the presence of aldehydes [81, 82]. By combining and optimizing a YqhC sensing module and a reporting module under the control of the *yqhD* promoter region, a bi-modular sensor system for in vivo detection of various aldehydes at the concentration range of 1–10 mM was constructed in *E. coli* [83]. This sensor system has been successfully employed for improving the in vivo production of glycolaldehyde but it was barely responsive to isobutyraldehyde. Since isobutyraldehyde and glycolaldehyde have minor differences in structures, it is highly expected to obtain a gain-of-function variant of YqhC for isobutyraldehyde detection and high-throughput screening, by either random mutagenesis-aided directed evolution or structure-based computational design (Fig. 3C). Successful examples of changing or expanding the specificity of existing sensors have been observed in many studies such as vanillin [84].

### Increasing production strain stability

Strain stability is a prerequisite for industrial production. Strains carrying multicopy plasmids are generally not applicable in the industry due to the instability and the uncontrollability of multicopy plasmids. In addition, multicopy plasmids require antibiotic resistance markers to be retained in the cell, which is not suitable for large scale fermentations. To obtain industrially applied strains, multicopy chromosomal integration of target pathway genes may be a solution. The CRISPR-Cas system allows the knock-in of heterologous genes, integration of large synthetic pathways as well as the combinatorial and multiplex modifications in chromosomes [85–89]. Using CRISPR-associated transposases (MUCICAT) and targeting the crRNA to multicopy loci of the *E. coli* genome, up to 10 copies of integration were achieved [90]. *E. coli* strain harboring these cargos exhibited a 2.6-fold increase in target protein expression than that carrying the conventional multicopy plasmid. Another multi-copy integration tool designated as gene drive delta site integration CRISPR system (GDi-CRISPR) was developed in *Saccharomyces cerevisiae* (*S. cerevisiae*) by placing the gRNA in the donor fragments and integrating into the genome [91]. This method increased the frequency of excision repair and enabled up to 6 copies of integration. It is highly worthy to evaluate the effect of ad vanced CRISPR-assisted integration techniques in establishing a high-performing strain for isobutyraldehyde production (Fig. 3D).

Considering the boiling point of isobutyraldehyde is not sufficiently low to allow for efficient stripping at the elevated bioreactor pressures that are required to allow for gas mass transfer, several aspects should be considered at fermentation process to reduce the toxicity on cells and product loss mediated by aldehydes reactivity. First, given that isobutyraldehyde is gradually oxidized to isobutyric acid, especially in the range of 30℃ and 50℃, the fermentation process should be conducted at 30℃ to minimize the oxidation effect. Second, the conversion of glucose to isobutyraldehyde leads to an imbalanced redox state. Per isobutyraldehyde generates an excess of NADH, which needs to be recycled through oxidative phosphorylation. In this regard, supplying oxygen (using a gas-stripping system) is beneficial for isobutyraldehyde production. To be noted, oxygen levels should be evaluated and carefully controlled during the fermentation process, because the supply of ample oxygen will result in oxidation of isobutyraldehyde to isobutyric acid. After gas-stripping extraction, an appreciable concentration of isobutyraldehyde remains in the broth, leading to cell toxicity and product loss. This might be relieved by replacing a certain amount of medium with fresh one intermittently or using a two-phase extraction strategy, keeping isobutyraldehyde at a relatively low level during long-term fermentation.

## In vitro framework converting C_1_ feedstocks to isobutyraldehyde

### Establishing a cell-free system for isobutyraldehyde synthesis

In general, in vivo synthesis faces a constant metabolic burden of producing target molecules; therefore, time- and labor-intensive efforts are required to achieve the flux balance between target pathways and nonessential ones while pushing toward high titers. In vitro platforms can address these challenges by exploring the capabilities outside the homeostatic ranges. For example, in vitro synthesis using the cell-free system removes the constraints of cell viability and the complexity of cellular organisms by constructing the biosynthetic pathways using crude cell lysates or purified enzymes. In contrast to a cell system that is highly sensitive to isobutyraldehyde toxicity presumably due to the loss of membrane integrity, the cell-free system has the potential to tolerate higher concentrations of isobutyraldehyde and the tolerance limit can be further addressed by the engineering of the respective enzymes. Although a reliable cell-free system converting inexpensive inputs to isobutyraldehyde has not yet been reported, the cell-free system producing isobutanol from glucose has been established and optimized. By designing a molecular rheostat for maintaining adequate ATP levels and improving the solvent tolerance of key enzymes in combination with eliminating the thermodynamic limitations and extracting the product continuously, the isobutanol production in the synthetic biochemistry system has reached an accumulated titer of 275 g L^−1^ within 5 days under bioreactor conditions, along with a maximum productivity of 4 g L^−1^ h^−1^ and 95% yield [30, 92]. The isobutanol synthetic cascade requires two equivalents of NAD(P)H, which can be generated via either the canonical glycolysis pathway or the modified non-phosphorylative Entner–Doudoroff-pathway (np-ED) [93]. Therefore, the self-regulatory system maintained redox neutrality during the long-term production of isobutanol. It should be noted that one equivalent of NAD(P)H is consumed during the conversion of isobutyraldehyde to isobutanol, thus the cofactor needs to be rebalanced via rewiring the pathways to achieve a stable synthesis of isobutyraldehyde.

### Cell-free synthesis of isobutyraldehyde from CO_2_

**Fig. 4:**
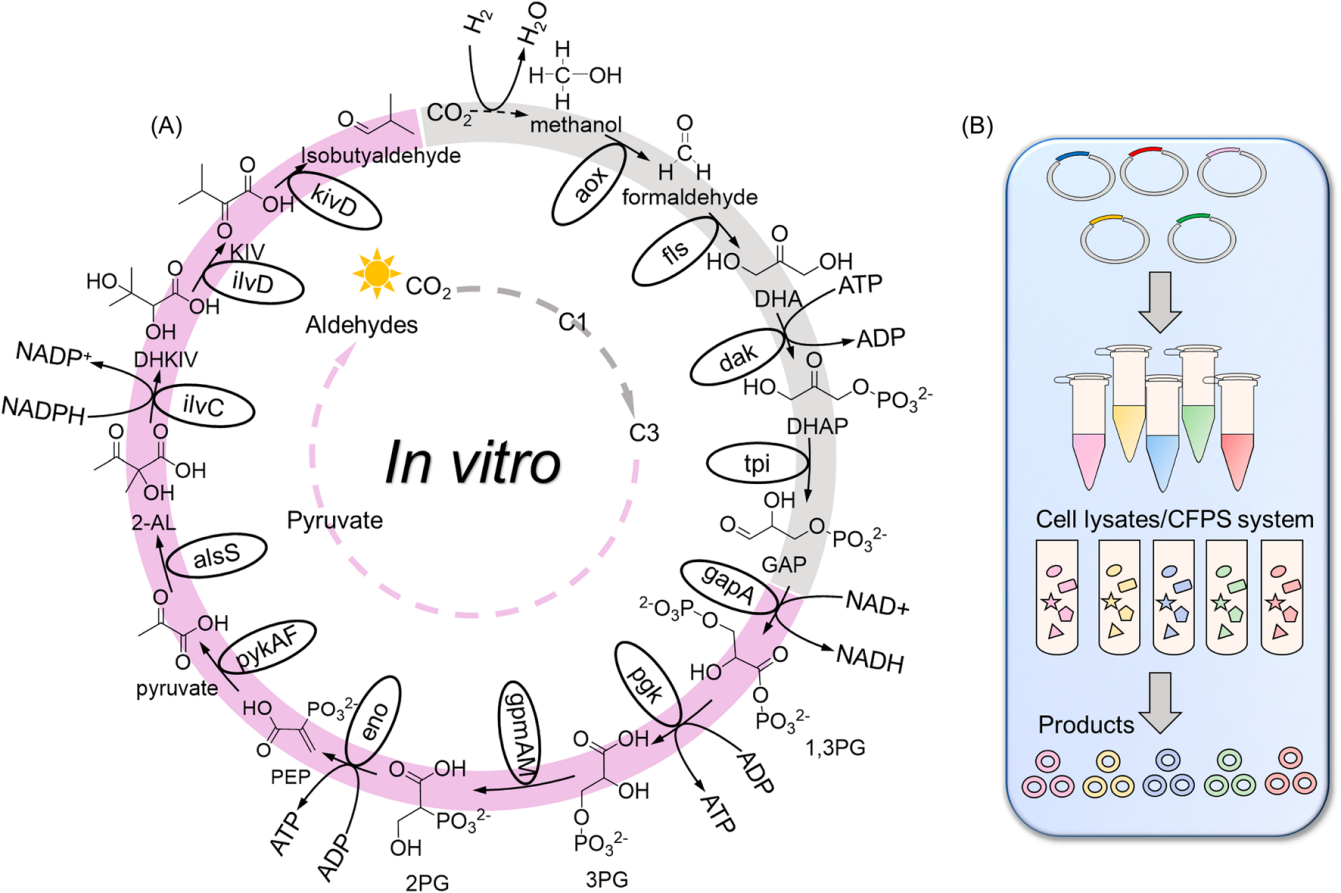
In vitro framework converting C_1_ feedstocks to isobutyraldehyde and other chemicals. **A** Design of the cell-free system converting CO_2_ to isobutyraldehyde. The proposed in vitro pathway for isobutyraldehyde production was assembled using two parts. The first one converting CO_2_ to D-glyceraldehyde-3-phosphate (GAP) was generated based on the first two modules of ASAP [13] and was indicated in grey color. The second one converting 3-phosphoglycerate to isobutyraldehyde was indicated in pink color. Metabolite: Dihydroxyacetone (DHA); Dihydroxyacetone phosphate (DHAP); Glyceraldehyde-3-phosphate (GAP); 1,3-Biphosphoglycerate (1,3PG); 3-Phosphoglycerate (3PG); 2-Phosphoglycerate (2PG); Phosphoenolpyruvate (PEP); 2-acetolactate (2-AL); 2,3-Dihydroxy-isovalerate (DHKIV); 2-Ketoisovalerate (KIV); Isobutyraldehyde (IBAL). Enzymes: alcohol oxidase (aox); formolase (fls); dihydroxyacetone kinase (dak); triosephosphate isomerase (tpi). **B** Cell-free framework for producing a wide array of chemicals. Similar to the in vitro system of isobutyraldehyde, cell lysates with pathway-specific enzymes or the designed cell-free protein synthesis (CFPS) system can be employed for production of various chemicals by assembling with the first two modules of ASAP

Cell-free biomanufacturing  carries high potential to maximize carbon conversion rate when using CO_2_ as a substrate [94, 95]. This can be facilitated by the implementation of redesigned CO_2_ fixation modules along with a balanced cofactor system. Establishing a cell-free system converting CO_2_ to isobutyraldehyde is interesting but also challenging. In a recent study, Cai et al. reported the cell-free synthesis of starch from CO_2_ by constructing an artificial starch anabolic pathway (ASAP), which produces C_1_ units via the chemical reduction catalyst and converts C_1_ units to starch via a chemoenzymatic process [13]. This system consisted of four modules and the module two achieved a substantially high yield of C_3_ compound, D-glyceraldehyde 3-phosphate (GAP). GAP acts as an important intermediate of the glycolysis pathway and can be easily converted to pyruvate by endogenous enzymes. Therefore, the first two modules of ASAP can be assembled either with cell lysates containing pathway-specific enzymes or the designed cell-free protein synthesis (CFPS) system and extended to the production of a wide array of chemicals including isobutyraldehyde (Fig. 4). Associated issues such as cofactor recycling and balancing still need to be addressed before the system be applied to a product of interest.

### Supplying cost-effective and renewable energy sources

The primary challenge toward the practical realization of cell-free C_1_-biomanufacturing is the supply of cost-effect energy sources. In the past decade, enormous efforts have been sought toward electrochemical conversion of CO_2_ [46]. Using widely available electrical energy, the CO_2_ reduction to CO along with byproduct hydrogen has been reported in technological readiness level, achieving high current densities, remarkable Faradaic efficiencies, and excellent process stabilities [96–98]. This allows CO and hydrogen to act as cost-effective, renewable reducing power for generating ATP and protons in techno-economical C_1_ fermentation. The supply of ATP and N AD(P)H for cell-free biosynthetic reactions can also be addressed by implementing energy regeneration systems using extract-based platforms [95]. For example, incorporating components from *Shewanella oneidensis* and other electrically active microbes into a cell-free system mediates the development of electrobiological machinery, which would utilize electrical energy to generate biological electron carriers [99]. Likewise, using synthetic vesicles comprising ATP synthase and bacteriorhodopsin, ATP can be sustainably generated from light [100]. These advances will significantly strengthen the economic viability of cell-free C_1_ biomanufacturing.

### Navigating enzymes with stable and reusable activity

A non-negligible challenge of the cell-free approach is the cost, which might be partially addressed using crude cell lysates instead of expensive purified enzymes and cofactors. However, a lysate-based cell-free system may be harnessed by the nonessential endogenous enzymes, leading to unwanted-byproducts formation and resources competition during in vitro conversion. One solution is to build the target pathways using thermostable enzymes, which allow the in vitro reaction to be operated at high temperatures and the endogenous metabolic enzymes to be inactivated [101]. To be noted, cofactor integrity also needs to be evaluated under the optimal conditions of enzymes. As a newly developed platform technology, CFPS allows the in vitro expression of target DNAs and rapid assembly of biosynthetic enzymes, thereby it can be streamlined for testing various enzyme combinations in a high-throughput manner [102]. To exploit suitable enzymes for robust synthesis of isobutyraldehyde, libraries of enzymes or mutants with high stability and activity as well as solvent tolerance could be screened simultaneously using CFPS technology in combination with biosensing modules. In addition to bio-discovery, developing a convenient and simple system for efficient cell-free synthesis is also important. Recently, a one-step self-assembly system based on CipA-scaffold was established for the simultaneous expression and immobilization of isobutyraldehyde biosynthetic enzymes. This strategy enhanced the thermostability of LeuDH and KivD and facilitated the in vitro conversion of valine to isobutyraldehyde with a higher conversion rate [19]. Such a CipA-scaffold could be employed for the cell-free conversion of C_1_ feedstock to isobutyraldehyde as well as other high-value chemicals. After lowering the cost of enzymes a nd co factors (via in situ recycling), a reliable cell-free system will be established for the large-scale production of isobutyraldehyde from inexpensive inputs.

## Conclusions and future perspectives

Oxo chemicals, including oxo aldehydes, oxo acids, and oxo alcohols, are used for the synthesis of a wide array of industrial and consumer products, including plasticizers, fine chemicals, and pharmaceuticals. The utilization of bio-oxo chemicals as the pr ecursors and intermediates of polymers and biofuels is one of the most recognized applications of synthetic biology [103, 104]. As a newly preferred feedstock in the biomanufacturing world, C_1_ feedstocks are gaining attention in the oxo chemicals industry. Converting C_1_ feedstocks to oxo chemicals avoids the release of greenhouse gas that is generated from the traditional chem-oxo process, meanwhile, recycles the existing greenhouse gas contributing to carbon neutrality.  Exemplifying isobutyraldehyde, its production from CO_2_ has achieved a benchmark productivity of 6.23 mg/L/h, which was 82% higher than the average productivity of microalgal biodiesel that was produced by well-designed production systems [18]. This starting point shows the potential of oxo aldehydes production from C_1_ feedstocks.

In the long term, it would be interesting and necessary to establish an economical bio-production system for converting C_1_ feedstocks to oxo chemicals. Among all the C_1_-pathways verified in vivo or in vitro, CBB cycle, WLP, and ASAP have been successfully exemplified for the production of bio-chemicals, while rGlyP, rTCA cycle, and 3-HP bicycle have been partially demonstrated. In this work, analysis of energy cost and enzyme numbers of the reassembled pathways suggested that WLP, rGlyP, and rTCA cycle may be the efficient ones showing high potential in isobutyraldehyde prod uction. Likewise, other oxo chemicals that derive either from pyruvate or acetyl-CoA can be expected to be produced from C_1_ feedstocks using these C_1_ pathways.

Scanning across the row of the same substrates in Table 1, it appears that the highest production values of oxo aldehydes are relatively lower than that of alcohols, partially due to their toxic and unstable properties. Taking isobutyraldehyde as an example, the highest productivity from glucose is only 0.29 g/L/h, while the productivity of isobutanol has reached 4 g/L/h using an in vitro system [19, 22]. Regarding the efficient production of downstream alcohols and the close relationship between aldehydes and alcohols, there is still a high possibility of reaching higher titers and productivities in aldehydes production if all the engineering improvements debottlenecking the limits were incorporated into the final integrated system. Beyond the production process, the industrial scale-up should involve an efficient downstream separation process. Exemplifying volatile isobutyraldehyde, the bio-oxo production system uses a gas-stripping process. When the gas is fed into the bioreactor, mass transfer of the air applie d into the bioreactor determines the stripping efficiency for isobutyraldehyde, thereby different bioreactor modules such as typical mechanically agitated fermenters and air-lift bubble column reactors could be utilized. Taken together, it is highly expected that the proposed bio-oxo technology using C_1_ feedstocks will be an attractive direction to the oxo chemicals manufacturing world.

## Data Availability

All data and materials in this study are included in this published article.
